# Before and during the COVID-19 Pandemic, Physical Fitness Association with Mental Health among Higher Education Students: A Multi-Group Analysis Model

**DOI:** 10.3390/ijerph192215393

**Published:** 2022-11-21

**Authors:** Ibrahim A. Elshaer, Mohamed A. Zayed

**Affiliations:** 1 Management Department, College of Business Administration, King Faisal University, Al-Hassa 31982, Saudi Arabia; 2 Faculty of Tourism and Hotel Management, Suez Canal University, Ismailia 41522, Egypt; 3 Deanship of Student Affairs, King Faisal University, Al-Hassa 31982, Saudi Arabia; 4 Department of Fitness, Gymnastics and Sports Show, Faculty of Physical Education Alexandria University, Alexandria 21625, Egypt

**Keywords:** physical fitness, COVID-19, mental health, depression, anxiety, stress, university students, multi-group analysis

## Abstract

The coronavirus disease 2019 (COVID-19) pandemic, caused by the severe acute respiratory syndrome coronavirus 2 (SARS-CoV-2), created a significant problem people’s health around the world. The mental and physical health of entire populations has been negatively impacted due to the introduction of several restriction methods. Maintaining a specific physical activity and fitness level is crucial given the pandemic situation. The connection between physical fitness and mental health has recently received growing attention. In contrast to the message from physiological research, which lauds the general benefits of fitness for physical health, the corresponding psychological literature reveals a more complex relationship. This paper outlines the research evidence, focusing on the relationship between physical fitness and depression, anxiety, and stress before and during the COVID-19 pandemic. Data were obtained from 390 higher education students (measuring their perception before and during the pandemic). They were analyzed by a structural equation modeling multi-group analysis to detect the variance in the test relationship before and during the COVID-19 pandemic. Theoretical and empirical implications are also discussed.

## 1. Introduction

Numerous restriction conditions that were introduced as a result of the spread of COVID-19 have affected people’s mental and physical health. As a result, sustaining a particular level of physical activity, exercise, and fitness is vital. The terms “Physical fitness”, “Physical activity”, and “Exercise” refer to distinct concepts [1,2,3,4]. However, they are frequently confused and occasionally used interchangeably. Physical activity is any movement of the skeletal muscles that usually results in energy expenditure and is commonly measured with kilocalories. Exercise is a subcategory of physical activity that is planned, structured, and repetitive, with the ultimate or intermediate goal of enhancing or maintaining physical fitness. Physical fitness (PF) is a set of attributes that are either health- or skill-related [5,6,7]. Physical fitness has been defined as the capacity to perform regular duties with intensity and alertness, without excessive fatigue, and with sufficient energy to enjoy leisure activities and respond to unforeseen emergencies [7,8,9].

Appropriate PA and PF preserve the immune system and improve a person’s overall quality of life and mental health [10,11,12]. It is possible to slow or stop the spread of infectious diseases caused by contagious viruses if one’s immune system is strengthened through appropriate exercise, PA, and PF [13]. The common tendency of shortened exercise, PA and PF levels, inactive lifestyle, and the applications of modern devices in everyone’s lives have driven the World Health Organization (WHO) to make suggestions on the level of adequate exercise, PA, and PF for different groups [14]. As a result of the COVID-19 pandemic, restrictive procedures have been implemented. These procedures involve keeping a social space between people and entirely or partially shutting down economic activities. This has contributed to the worldwide trend of reduced exercise, PA, and PF [15,16,17]. University students are not an exception; they are one of the social groups whose mental and physical health are negatively impacted by the COVID-19 pandemic [18,19,20]. Comparing the years after the emergence of COVID-19 to those before, a large number of studies from all over the world point to a significant decline in student PF of anywhere between 48% and 61% [18,19,20,21]. Even before the COVID-19 pandemic, studies on the quality of life of university students indicated the presence of some mental disorders, such as depression, anxiety, and stress [22].

On the other hand, a higher PF level can result in a decreased feeling of stress [23]. A reduction in stress resulting from adequate PF practices is one factor that may contribute to an enhancement in psycho-physical disorders [24]. The COVID-19 pandemic negatively impacted the mental health of students due to the introduction of partial or complete lockdowns. Theoretically, a poor psycho-physical situation leads to harmful side effects, such as high stress levels, anxiety, and depression [25,26,27]. Unfortunately, while the physical benefits associated with PF are well documented, there is scarce empirical evidence supporting an equivalent relationship between PF and psychological well-being. This study aims to provide empirical evidence that tests the association between PF and mental health dimensions such as depression, anxiety, and stress, as well as to compare the results before and during the COVID-19 pandemic. To the author’s knowledge, this is one of the first studies investigating such relationships before and during the COVID-19 pandemic on a sample of university students in the Kingdom of Saudi Arabia (KSA).

### 1.1. PF and Depression

In his study, Martinsen [28] examined the literature regarding the effect of PF on people with depression. Initially, he discovered that such individuals tended to be physically inactive and had a lower physical work capacity when compared to the whole population. This finding rapidly contended for incorporating physical fitness training into a comprehensive depression treatment program, while simultaneously indicating the potential challenges of implementing an exercise program with a population that is not predisposed. Even though many studies emphasize the importance of using PF to treat depression, Martinsen [28] discovered that the antidepressant effect of non-aerobic exercise was equivalent to that of aerobic exercise. Those who continued to exercise regularly following the conclusion of a one-year training program had lower levels of depression than those who were sedentary. According to Martinsen, people ranked PF as “the most important element in a comprehensive treatment program for depression”. Based on the most recent literature [17,20,22], it appears reasonable to assume that PF programs may reduce feelings of depression. Therefore, we propose the following hypothesis:

**Hypothesis 1 (H1).**  *There are statistically significant differences in the relationship between PF and depression (as a mental health dimension) before and during the COVID-19 pandemic.*

### 1.2. PF and Anxiety

Several studies examined the link between PF and anxiety. Most of these studies concluded that there is a reliable link between PF and anxiety reduction. This was true regardless of the anxiety measures used (trait behavioral, physiological, or self-reported) [29]. In addition, a meta-analysis conducted by Long [30] specifically examined studies that differentiated between people coping with stress and those who do not conclude that exercise and PF training programs effectively reduce anxiety. This assumption was especially true for people who were experiencing chronic work stress. The previous results were comparable to those found in other exercise-stress meta-analyses and anxiety-reduction treatments [31]. It is reasonable to assume that most research studies have focused on aerobic exercise. A scant number of studies investigating non-aerobic activities, such as strength and flexibility training, have shown marginally lower anxiety levels [32,33,34]. Although further research is needed, it does appear that PF needs further research to investigate its association with anxiety reduction. Therefore, we propose the below hypothesis:

**Hypothesis 2 (H2).**  *There are statistically significant differences in the relationship between PF and anxiety (as a mental health dimension) before and during the COVID-19 pandemic.*

### 1.3. PF and Stress

In several recent studies, the potential protective effects of PF against stress have been examined [35,36,37]. Nevertheless, whether this effect should be classified as psychological or physiological is debatable. Aside from this, the research suggests that improvements in physical condition or fitness are likely to facilitate people’s ability to deal with stress [38]. Reviewing the work of Fillingim (1993) [39], a distinction has been drawn between research that is either cross-sectional (classifying participants as “fit” or “unfit” and then observing differences between the groups) or longitudinal (using training and control groups), depending on the design of the study. The findings obtained from both methods can be summed up as inconclusive; however, most of them indicate that a higher level of physical fitness is associated with a lower stress level. In addition, some studies have concluded that PF practices do, in fact, illustrate how people react to stress [40,41,42]. In each of these, distinctions have been developed between aerobic exercise and anaerobic strength training. The study participants typically worked out for 12 weeks at least three times per week at a moderate intensity. In conclusion, PF may show a reduced psychosocial stress response, but exercise’s role is likely preventative rather than corrective, and the stress response remains partially understood. Therefore, we propose the below hypothesis:

**Hypothesis 3 (H3).**  *There are statistically significant differences in the relationship between PF and stress (as a mental health dimension) before and during the COVID-19 pandemic.*

In addition to the previous introduction and the hypotheses’ development section, this paper was designed to include the employed research methodology with the sampling selection and measurement development subsections, followed by the results of the data analysis. The discussion and implications for policymakers and scholars’ roles were then illustrated. Finally, the conclusion section was elaborated.

## 2. Methods

A quantitative cross-sectional research design was adopted to serve the purpose of the current study, in which previous literature was extensively reviewed to develop the study measures and construct the research framework. Consequently, the research hypotheses were formulated. Soon after, primary empirical data were collected using an online questionnaire, and the collected data were analyzed by using the SEM multi-group analysis approach.

### 2.1. Sampling Selection

The study population includes all higher education students enrolled in three public universities in the Kingdom of Saudi Arabia (KSA): King Faisal University, Umm Al-Qura University, and Imam Abdulrahman bin Faisal University. According to each university website, the total number of students enrolled in 2021 was around 117,000. Due to the restrictions imposed by the lockdown, these universities were among those compelled to switch to using online platforms during the pandemic.

The questionnaire was distributed to the students who were the focus of the study through personal relationships and networks, such as lecturers and professors at the participating universities. They were instructed to send the survey link to as many people as possible through WhatsApp or e-mail. Students were given the option of participating in an anonymous survey or not.

All information that could be used to identify individual identity was eliminated from the survey’s findings to protect the participants’ privacy. Participation in the survey was completely voluntary and anonymous. Questions regarding the student’s name, age, and the institution’s name were optional. A total of 400 surveys were distributed, and a response rate of 97% was achieved with 390 valid questionnaires. The current study sample size of 390 responses is proper and adequate to be analyzed with SEM, as it fulfils Nunnally’s [43] advice of at least ten responses for each scale item (the current study had 30 scale items, producing a recommended minimum sample size at least 300); it also conformed with Hair et al.’s [43] criteria of at least 100–150 answers to generate adequate estimations. Furthermore, as proposed by Krejcie and Morgan [44], when the total size of the population surpasses 1,000,000, the smallest sufficient sample size would be at least 384 replies. Considering all previous arguments, the current sample size of 390 is proper and adequate for further data analysis.

In September and October 2022 (when the students returned to the typical traditional classroom), the questionnaire was made available. The students were asked to evaluate their perception of the same questions before and during the COVID-19 pandemic.

### 2.2. Measures and Instrument Development

The standard psychometric properties were used to assess and evaluate the literature and select the research scale. Student mental health was measured by utilizing the DASS-21 (depression, anxiety, and stress) scale. This measure has 21 items and is simple to use in clinical and non-clinical research; it is employed to detect the negative emotions experienced by individuals [45]. Each dimension (depression, anxiety, and stress) of the DASS-21 has seven variables.

The participants were asked to indicate the degree to which the item applied to them before and during the COVID-19 pandemic. Similarly, the perceived physical fitness scale developed by Abadie [46] was employed in the current study to measure student physical fitness (PF) before and during the COVID-19 pandemic. The PF scale focuses on the different characteristics of student physical fitness, such as muscular strength (i.e., “an object that I can lift once with slight difficulty soon becomes strenuous when I attempt to lift it repeatedly”; flexibility (i.e., “I possess greater muscular flexibility than most individuals my age”); body composition (i.e., “I do not need to alter my weight to improve my physical health”); and cardiorespiratory fitness (i.e., “I am better able to walk briskly for twenty minutes than most individuals my age)”.

The scale employs a four-level Likert grading structure with 1 to 4 values, where (1) indicates “did not apply to me at all”, (2) refers to “applied to me to some degree, or some of the time”, (3) implies “applied to me to a considerable degree, or a good part of the time, and (4) indicates “applied to me very much, or most of the time”.

We designed the self-administrated online survey to meet previous suggestions in the literature [47]. After creating the measurement items, one of the research team entered the questions on Google Forms; later, the research team reviewed it before sending the Uniform Resource Locator (URL) to the participants. The research aims and objectives were clearly stated in the questionnaire introduction, and the targeted students were invited to complete the survey. The participants received the survey link (written in both English and Arabic) via formal university e-mails. Daily, the entire research team monitored the responses. The participant’s optional personal information (name, phone number, e-mail address, and social media profiles) was located at the end of the questionnaire.

Fourteen senior students and fifteen academic professors were invited to evaluate the study scale for clarity, simplicity, and appropriateness. During this process, no substantial changes were detected; however, a few amendments were observed to improve the scale’s language. To assess the reliability of the scale items, Cronbach’s alpha (a) values were estimated. The alpha (a) values were found to be between 0.93 and 0.96, surpassing the proposed cut-off value [48].

As we employed a self-reported online survey, common method variance (CMV) was a potential a problem [49]. Therefore, we adopted three techniques to deal with this. (1) The dependent questions (mental health dimensions) were structured in the survey to come before the independent questions (physical fitness). (2) The respondents’ personal information was kept confidential. (3) We used Harman’s single-factor method, in which all of the employed questions were subjected to an exploratory factor analysis (EFA) in the SPSS software, with the restriction that only one factor should be retrieved without rotating the data. This was done so we could adhere to the limitation that only one factor should be found. The investigation results showed that CMV was never an issue at any point during our inquiry because only one variable accounted for 42% of the variance in the data [50].

### 2.3. Methods of Data Analysis

In this research paper, three successive stages of data analysis were conducted. The descriptive data analysis was conducted in the first stage by employing the “IBM Statistical Package for Social Sciences” (SPSS) version 24 to evaluate the participants’ characteristics, the data mean, and the standard deviation as a basis for the next stage: multivariate data analysis. The multivariate data analysis was employed, using structural equation modeling (SEM) and the Amos program, to evaluate the scale validity and to test the study hypothesis. Finally, in the last stage (stage three), the multi-group analysis technique was implemented in SEM to detect the variance between the two groups of interest (before and during the COVID-19 pandemic). Several SEM goodness of fit (GOF) criteria were employed following recommendations from [51,52,53], as depicted in Table 1.

## 3. Results

### 3.1. Stage One: Results of Descriptive Analysis

The study sample included 390 students from KSA’s three public universities. The participants from King Faisal University represent (*n* = 152; 39%), Umm Al-Qura University (*n* = 121; 31%), and Imam Abdulrahman bin Faisal University (*n* = 118; 30%). Regarding their sociodemographic profiles, the participants were primarily male (*n* = 270; 69%), and the majority were between the ages of 17 and 26 (*n* = 330; 84%). A little less than one-quarter of the students were working students (*n* = 94; 24%). Most were enrolled in bachelor’s degree programs (*n* = 300; 76%), whereas the vast majority were in their fourth year (*n* = 199; 51%), followed by third-year students (*n* = 140; 36%). The majority were students at business school (*n* = 156; 40%), followed by medical and health faculties (*n* = 129; 33%), and engineering students (*n* = 105; 27%).

The values for the mean ranged anywhere between 2.22 and 3.15, while the values for the standard deviation were between 0.876 and 1.05. As a direct result, the data were found to be more dispersed and less concentrated in the center [54]. Additionally, the virtual observation of the skewness and kurtosis scores (data distribution) indicated no values that surpassed −2 or +2, giving evidence of normal normality distribution (univariate) [55]. Using an independent t-test sample method, the mean of the early and late responses was determined. Non-response bias was not an issue in our study because no statistically significant differences were observed [54].

### 3.2. Stage Two: Results of Multivariate Analysis

We performed a two-step sequential structural equation modeling (SEM) methodology, as suggested by [56]. In the first step (Step 1), the scale was evaluated against validity and reliability criteria. For this purpose, we performed two first-order CFA models (one CFA for the group of data before the COVID-19 pandemic and the other CFA for the group of data during the COVID-19 pandemic). Amos v24 with a maximum likelihood estimation (ML) approach was adopted. In the second step (step 2), the nomological model (structural) was assessed to evaluate the research hypotheses in the two models of interest (before and during the COVID-19 pandemic). All prerequisites for running a CFA and SEM were fulfilled. These prerequisites include conditions pertaining to the sample size, missing data, outliers, multicollinearity, and normality.

### 3.3. Step 1: First-Order CFA Models for Construct Validity and Reliability

To evaluate the discriminant and convergent validity of the scale used in our study, two first-order CFA models (before and during COVID-19 models) were drafted, and estimates were calculated in Amos v24. The model has four latent factors with 30 reflective items (nine reflective items measure physical fitness, seven measure depression, seven measure anxiety, and seven measure stress). The GoF indices demonstrated a good model fit to the data in the two models, as depicted in Table 1. The four employed factors’ composite reliability (CR) scores in the two models of interest exhibited excellent internal consistency (see Table 1), as they ranged between 0.939 and 0.985, and thus, surpassed the proposed limit value of 0.70 [57]. The factors’ reliability was further confirmed by calculating Cronbach’s alpha values, which were all found to have values more than the critical threshold points of 0.70, as shown in Table 1 [49]. Moreover, Table 1 provides more evidence that guarantees the employed scale convergent validity, whereas all factors have high significant loadings values (FL > 0.7, *p* < 0.001) in the two models. The average variance extracted (AVE) values for all four employed scales surpassed the threshold point [58], which provides more evidence to support convergent validity. Additionally, as noted in Table 1, the maximum shared variance (MSV) values for each of the study measures are below the corresponding AVE values, exhibiting adequate scale discriminant validity [51]. Lastly, as displayed in Table 2 the (bold values) are higher than the intercorrelation (below bold variables), presenting additional evidence to support the claim that the scale has sufficient discriminant validity [49].

### 3.4. Step 2: Hypotheses Testing in the Structural Models

After extensively reviewing the pertinent prior research, a specific theoretical model was developed to serve the purpose of the current study. Subsequently, empirical data were collected and analyzed to test whether they fit the proposed theoretical model [59,60]. The data were either approved or rejected according to how well the proposed model fit the data.

**Figure 1:**
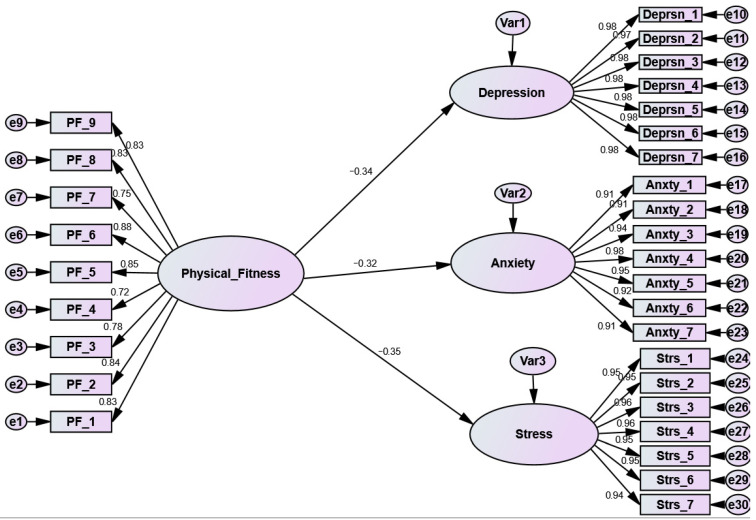
The structural model results before the COVID-19 pandemic.

**Figure 2:**
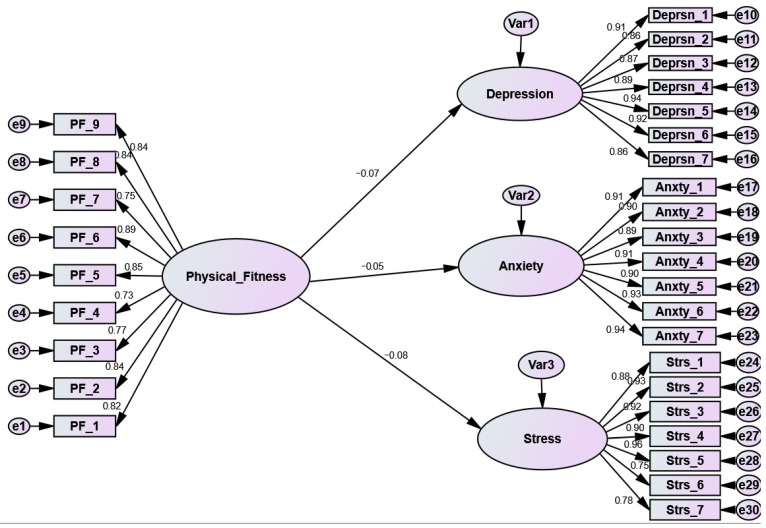
The structural model results during the COVID-19 pandemic.

Figure 1 and Figure 2 and Table 3 reveal the GoF of the two assumed and assessed models: before (Figure 1) and during (Figure 2) the COVID-19 pandemic. The chi-square (X^2^) GoF index was found to be significant in both models (*p* > 0.01); unlike another test, this significant *p*-value indicates that the null hypothesis (models is fit) was not supported. The significant x^2^ implies that (S) did not match (∑k). In other words, the covariance matrix (Actual) did not match the estimate. The X^2^ in most SEM models is significant. However, the researcher cannot solely rely on that value to determine the model fit, as the X^2^ values are regularly affected by the sample size. Therefore, other GoF indices should be calculated and checked, such as the “Comparative Fit Index” (CFI) and “Root Mean-Square Error Approximation” [51,52,53]. As displayed in Table 2, model 1 (before the COVID-19 pandemic) exhibited slightly superior GoF indices than model 2 (during the COVID-19 pandemic). In general, the two models fit the data adequately.

Before the COVID-19 pandemic, all the proposed hypotheses were found to be significant with a negative path coefficient. More specifically, the link between PF and depression (as a dimension of mental health) was found to be significant and negative (β = −0.34, *t*-value = −6.620, *p* < 0.001). Similarly, the association between PF and anxiety (as a dimension of mental health) was found to be significant and negative (β = −0.32, *t*-value = −6.123, *p* < 0.001). Likewise, PF is significantly and negatively associated with stress as a mental health dimension (β = −0.35, *t*-value = −6.939, *p* < 0.001). According to the previous findings, when the students’ level of physical fitness increases, their negative emotions regarding mental health (depression, anxiety, and stress) decrease.

On the other hand, during the COVID-19 pandemic model and due to lockdown restrictions, PF failed to decrease the negative emotions of mental health (depression, anxiety, and stress. More specifically, the association between PF and depression (as a mental health dimension) was negative but insignificant (β = −0.07, *t*-value = −1340, *p* = 0.18). Similarly, the link between PF and anxiety (as a dimension of mental health) was found to be negative but insignificant (β = −0.05, *t*-value = −0.892, *p* = 0.362). Likewise, stress PF showed an insignificant negative relationship with stress as a dimension of mental health (β = −0.08, *t*-value = −1.493, *p* = 0.135

### 3.5. Stage Three: Multi-Group Analysis Results

To determine if the association between PF and the mental health (depression, anxiety, and stress) of college students differed before and during the COVID-19 pandemic (models 1 and 2), the structural model (regression weights) for the two sets of data were compared (i.e., variance). The exact structure of model 1 and model 2 were compared in the SEM multi-group analysis to detect any significant variation in the model path coefficients. The difference in the whole structure model in the two groups of interest can be detected by comparing the Chi-square (X^2^) values in the two models. When the X^2^ value of the baseline unconstrained structural model and the constrained (constrained path coefficients values to equal 1) model are compared, a significant difference is revealed between them, with a *p*-value that is less than 0.001. Consequently, the findings imply that either one or more of the path coefficients are not equivalent [60,61].

As pictured in Figure 1 and displayed in Table 3, all the path coefficients in model 1 (before the COVID-19 pandemic) were found to be negative and significant, while the results in model 2 (during the COVID-19 pandemic) were found to be negative but insignificant. Furthermore, according to the Amos results, the GoF for model 1 was higher than the GoF for model 2.

More specifically, in model 1 (before the pandemic), PF was able to decrease the negative emotions of depression (β = −0.34, *p* < 0.001) (H1), while in model 2, PF failed to reduce the negative feelings of depression among students (β = −0.07, *p* = 0.18). Likewise, PF before the pandemic (model 1) decreased the negative sense of anxiety (β = −0.32, *p* < 0.001), while during the pandemic, PF did not succeed in doing the same (β = −0.05, *p* = 0.362) (H2). Finally, PF successfully decreased stress among university students before the COVID-19 pandemic (β = −0.35, *p* < 0.001), while in model 2, PF failed to have the same association (β = −0.08, *p* = 0.135), (H3).

## 4. Discussion and Implications

The findings of this study spotlight several results that are relevant for both before and during the COVID-19 pandemic in the KSA context: (i) According to the findings, there were significant differences in the students’ levels of physical fitness both before and during the pandemic. (ii) During the pandemic, a significant association was found to exist between lower levels of physical fitness and higher levels of negative emotional states. (iii) Before the pandemic, increased levels of PF helped reduce the prevalence of unfavorable emotional states among university students. (iv) During the pandemic, there were indications that the percentage of college students who exhibited symptoms of anxiety, depression, and stress was much higher than before the pandemic.

The findings are consistent with the conclusions reached in earlier research, and the employed DASS-21 shows a high convergent and discriminant validity in measuring the university students’ levels of anxiety and depression. Similarly, the Perceived Physical Fitness Scale (PPFS) developed by Abadie [46] showed high reliability and validity in measuring the level of physical fitness among university students.

To the author’s knowledge, this study is among the first to evaluate the link between PF and student mental health (depression, anxiety, and stress) among university students in the KSA context. After returning to the normal classroom teaching process, the students were asked to evaluate their perception of the tested relationships before and during the COVID-19 pandemic. University students have been severely affected by the pandemic, which had a direct relationship with an increase in feelings of depression, anxiety, and stress [61].

The current study noted that during the COVID-19 pandemic, university students suffered more depression, anxiety, and stress than before the pandemic. However, the exerted level of PF can mitigate the negative emotions of mental health dimensions (depression, anxiety, and stress), as the result of the current study provides evidence that physical fitness reduced the negative emotions related to mental health before the pandemic but failed to do so during the pandemic due to lockdown and decreased PF practices among university students. These results are consistent with Schlichtiger [62] and Maher [63], who affirmed that a lower level of physical fitness is a risk factor for experiencing higher levels of mental stress. This became abundantly clear during the COVID-19 lockdown, when physical fitness, exercise, and sports opportunities were severely restricted.

Previous research has uncovered several stressors contributing to increased stress, anxiety, and depressive symptoms among university students [13,23,24,29,64,65]. Despite this, the COVID-19 pandemic is linked to a few novel risk factors that have rarely been described.

At the same time, during the pandemic, university students—and senior students, in particular—may experience potential job instability due to layoffs and potential crises and closures of workplaces [66,67,68]. Hence, senior students are expected to have higher levels of negative affective conditions than other groups [69,70,71]. During the pandemic, university students exhibited significantly higher levels of all three unfavorable emotional states of mental health compared to their levels before the pandemic, as indicated by the findings of the PPFS and DASS-21 scales. Moreover, the level of exerted PF among university students was higher before the pandemic than during the pandemic. These results are in line with [72], whose findings showed decreased physical fitness, academic performance, and overall quality of life among university students during the COVID-19 pandemic.

According to the findings of the current study, university students faced an insufficient level of PF and an elevated threat of experiencing psychological distress (depression, anxiety, stress) during the COVID-19 pandemic; therefore, policymakers need to make greater and more consistent efforts to improve students’ positive mental health and overall well-being. Colleges and universities ought to provide sufficient areas for students to engage in physical fitness activities, in addition to providing early detection and prevention-tailored programs for individuals suffering from mental health disorders. Additionally, policymakers should design these tailored programs before students graduate. Students should participate in treatments for depression, stress, and anxiety because these conditions may impact their future careers as healthcare professionals.

The study has some shortcomings, the vast majority of which are likely to be addressed by additional research in the future. This study evaluated students’ experience after returning to the regular classroom to investigate the association between PF and mental health before and during the COVID-19 pandemic. However, several other factors, such as gender, the support of family members, and the year studied, may act as mediators and have an association with the tested relationships. Future research should investigate a broader range of factors that affect students’ mental health, such as the learning process. Cross-sectional data make it impossible to determine causal relationships between variables, which further limits the generalization of the current study results. Even though we avoided CMV per Podsakoff’s [73] suggestions, future researchers may choose to support this study’s model with longitudinal data (before, during, and after the pandemic) or a combination of data sources. Using a multi-group analysis, the suggested research model can be tested in a different context (country and/or industry) to verify or falsify the current study results [74].

## 5. Conclusions

The study investigated the association between PF and university students’ mental health before and during the COVID-19 pandemic. A self-reported online questionnaire was used to collect the data for this study, and 390 students responded. These students were asked the same questions both before and during the pandemic. A SEM multi-group analysis and the Amos program version 24 were used to analyze the data that were obtained. Before and during the pandemic, the results of the SEM indicated that there were significant differences in the students’ levels of physical fitness. Similarly, during the pandemic, a significant association between lower levels of physical fitness and higher levels of negative emotional states was discovered. In addition, prior to the pandemic, increased levels of PF helped reduce the prevalence of negative emotional states among university students. In the midst of the pandemic, the proportion of college students exhibiting symptoms of anxiety, depression, and stress was significantly higher than that prior to the pandemic. Further research can employ additional mediating factors (gender, family support) and a multi-group analysis to test the proposed model in three different groups (before, during, and after the pandemic) or in different industrial contexts. To reduce the pandemic’s negative effects, it is important to monitor and promote students’ mental health and increase PF-friendly university spaces.

## Figures and Tables

**Table 1:** Psychometric properties in the two comparative models.

		**Before the Pandemic Model**					**During the Pandemic Model**				
**Study Dimensions**	**Items’ Abbreviations**	**SFL**	**α**	**CR**	**AVE**	**MSV**	**SFL**	**α**	**CR**	**AVE**	**MSV**
**Threshold Value**		**>0.7**	**>0.7**	**>0.7**	**>0.5**	**>AVE**	**>0.7**	**>0.7**	**>0.7**	**>0.5**	**>AVE**
Physical Fitness (PF)	PA_1	0.821	0.943	0.947	0.665	0.104	0.815	0.931	0.947	0.665	0.006
	PA_2	0.839					0.834				
	PA_3	0.778					0.771				
	PA_4	0.728					0.730				
	PA_5	0.848					0.848				
	PA_6	0.886					0.888				
	PA_7	0.746					0.747				
	PA_8	0.840					0.845				
	PA_9	0.977					0.845				
Depression (Deprsn)	Deprsn_1	0.972	0.961	0.904	0.957	0.213	0.951	0.951	0.963	0.786	0.195
	Deprsn_2	0.980					0.835				
	Deprsn_3	0.981					0.863				
	Deprsn_4	0.981					0.862				
	Deprsn_5	0.978					0.937				
	Deprsn_6	0.979					0.916				
	Deprsn_7	0.977					0.836				
Anxiety (Anxty)	Anxty_1	0.930	0.951	0.977	0.861	0.226	0.890	0.941	0.971	0.828	0.195
	Anxty_2	0.899					0.896				
	Anxty_3	0.909					0.898				
	Anxty_4	0.953					0.907				
	Anxty_5	0.971					0.908				
	Anxty_6	0.927					0.937				
	Anxty_7	0.903					0.933				
Stress (Strs)	Strs_1	0.956	0.939	0.985	0.902	0.226	0.884	0.931	0.959	0.771	0.108
	Strs_2	0.955					0.928				
	Strs_3	0.955					0.917				
	Strs_4	0.952					0.896				
	Strs_5	0.947					0.963				
	Strs_6	0.944					0.754				
	Strs_7	0.939					0.783				

Before the pandemic model: χ^2^ (399, *n* = 390) = 860.244, *p* < 0.001, normed χ^2^ = 2.156, Standardized Root Mean Squared Residual (SRMR) = 0.020, Root Mean Square Error of Approximation (RMSEA) = 0.031, Comparative Fit Index (CFI) = 0.944, Normed Fit Index (NFI) = 0.933, Tucker-Lewis index (TLI) = 0.921, Parsimony Normed Fix Index (PNFI) = 0.673, and Parsimony Comparative Fix Index (PCFI) = 0.682. During the pandemic model: χ^2^ (399, *n* = 390) = 1284.381, *p* < 0.001, normed χ^2^ = 3.219, SRMR = 0.026, RMSEA = 0.039, CFI = 0.962, NFI = 0.945, TLI = 0.941, PNFI = 0.683, and PCFI = 0.699 Note: SFL = Standardized Factor Loading; α = alpha value; CR = Composite Reliability; MSV = Maximum Shared Variance; AVE = Average Variance Extracted. The threshold value was adopted from [51,52,53].

**Table 2:** Discriminant validity CFA results.

	Before COVID-19 Model				During COVID-19 Model			
	Anxty	PF	Deprsn	Deprsn	Anxty	PF	Deprsn	Deprsn
Anxty	**0.928**				**0.910**			
PF	−0.283	**0.815**			−0.043	**0.815**		
Deprsn	0.438	−0.304	**0.978**		0.442	−0.051	**0.887**	
Deprsn	0.475	−0.322	0.462	**0.950**	0.253	−0.076	0.328	**0.878**

Bold values: AVE squared root.

**Table 3:** Hypotheses testing results for before and during COVID-19 models.

		Before Pandemic			During Pandemic		
Hypotheses		Β-Value	*t*-Value	Results	Β-Value	*t*-Value	Results
H1	Physical Fitness → Depression	−0.34 ***	−6.620	Confirmed	−0.07 ^ns^	−1.340	Confirmed
H2	Physical Fitness → Anxiety	−0.32 ***	−6.123	Confirmed	−0.05 ^ns^	−0.892	Confirmed
H3	Physical Fitness → Stress	−0.35 ***	−6.939	Confirmed	−0.08 ^ns^	−1.493	Confirmed

Before Pandemic Model: “χ^2^ (402, *n* = 390) = 846.21.071, *p* < 0.001, normed χ^2^ = 2.105, SRMR = 0.029, RMSEA = 0.033, CFI = 0.983, NFI = 0.973, TLI = 0.957, PNFI = 0.622, and PCFI = 0.631)”. During Pandemic Model: “χ^2^ (402, *n* = 390) = 1558.554, *p* < 0.001, normed χ^2^ = 3.877, SRMR = 0.045, RMSEA = 0.075, CFI = 0.921, NFI = 0.907, TLI = 0.911, PNFI = 0.651, and PCFI = 0.666). *** *p*-value less than 0.001; ns: non-significant.

## Data Availability

Data are available upon request from researchers who meet the eligibility criteria. Kindly contact the first author privately through e-mail.

## References

[1] Caspersen C.J., Powell K.E., Christenson G.M. (1985). Physical Activity, Exercise, and Physical Fitness: Definitions and Distinctions for Health-Related Research. Public Health Rep..

[2] Vanhees L., Lefevre J., Philippaerts R., Martens M., Huygens W., Troosters T., Beunen G. (2005). How to Assess Physical Activity? How to Assess Physical Fitness?. Eur. J. Cardiovasc. Prev. Rehabil..

[3] Namasivayam V., Lim S. (2017). Recent Advances in the Link between Physical Activity, Sedentary Behavior, Physical Fitness, and Colorectal Cancer. F1000Research.

[4] EBSCOhost|20717853|Competitive and Cooperative Physical Fitness Training Programs for Girls: Effects on Physical Fitness and Multidimensional Self-Concepts. https://web.p.ebscohost.com/abstract?direct=true&profile=ehost&scope=site&authtype=crawler&jrnl=08952779&asa=Y&AN=20717853&h=VPJKgAsuGiFkhI%2bgfB22foxXDL4FOj%2bQ0rxbhE298GYfZnW%2fZ9UIigGFEFKIAgErne2QZel7CU9dLTJ%2bXhQjuQ%3d%3d&crl=c&resultNs=AdminWebAuth&resultLocal=ErrCrlNotAuth&crlhashurl=login.aspx%3fdirect%3dtrue%26profile%3dehost%26scope%3dsite%26authtype%3dcrawler%26jrnl%3d08952779%26asa%3dY%26AN%3d20717853.

[5] Chen W., Hammond-Bennett A., Hypnar A., Mason S. (2018). Health-Related Physical Fitness and Physical Activity in Elementary School Students. BMC Public Health.

[6] Corbin C.B., Pangrazi R.P., Franks B.D. (2000). Definitions: Health, Fitness, and Physical Activity.

[7] Borremans E., Rintala P., McCubbin J.A. (2010). Physical Fitness and Physical Activity in Adolescents with Asperger Syndrome: A Comparative Study. Adapt. Phys. Act. Q..

[8] Henning L., Dreiskämper D., Tietjens M. (2022). The Interplay of Actual and Perceived Physical Fitness in Children: Effects on Motivation and Physical Activity. Psychol. Sport Exerc..

[9] Hernández-Jaña S., Escobar-Gómez D., Cristi-Montero C., Castro-Piñero J., Rodríguez-Rodríguez F. (2022). Changes in Active Behaviours, Physical Activity, Sedentary Time, and Physical Fitness in Chilean Parents during the COVID-19 Pandemic: A Retrospective Study. Int. J. Environ. Res. Public. Health.

[10] Stubbs B., Vancampfort D., Hallgren M., Firth J., Veronese N., Solmi M., Brand S., Cordes J., Malchow B., Gerber M. (2018). EPA Guidance on Physical Activity as a Treatment for Severe Mental Illness: A Meta-Review of the Evidence and Position Statement from the European Psychiatric Association (EPA), Supported by the International Organization of Physical Therapists in Mental Health (IOPTMH). Eur. Psychiatry.

[11] Boreham C.A.G., Kennedy R.A., Murphy M.H., Tully M., Wallace W.F.M., Young I. (2005). Training Effects of Short Bouts of Stair Climbing on Cardiorespiratory Fitness, Blood Lipids, and Homocysteine in Sedentary Young Women. Br. J. Sports Med..

[12] Ubaida-Mohien C., Gonzalez-Freire M., Lyashkov A., Moaddel R., Chia C.W., Simonsick E.M., Sen R., Ferrucci L. (2019). Physical Activity Associated Proteomics of Skeletal Muscle: Being Proteomics of Skeletal Muscle: Being Physically Active in Daily Life May Protect Skeletal Muscle from Aging. Front. Physiol..

[13] Frontiers|Debunking the Myth of Exercise-Induced Immune Suppression: Redefining the Impact of Exercise on Immunological Health Across the Lifespan. https://www.frontiersin.org/articles/10.3389/fimmu.2018.00648/full?utm_source=vuanem.com.

[14] World Health Organization 2020 Guidelines on Physical Activity and Sedentary Behaviour|British Journal of Sports Medicine. https://bjsm.bmj.com/content/54/24/1451?s=09&int_source=trendmd&int_medium=cpc&int_campaign=usage-042019.

[15] Ammar A., Brach M., Trabelsi K., Chtourou H., Boukhris O., Masmoudi L., Bouaziz B., Bentlage E., How D., Ahmed M. (2020). Effects of COVID-19 Home Confinement on Eating Behaviour and Physical Activity: Results of the ECLB-COVID19 International Online Survey. Nutrients.

[16] Meyer J., McDowell C., Lansing J., Brower C., Smith L., Tully M., Herring M. (2020). Changes in Physical Activity and Sedentary Behavior in Response to COVID-19 and Their Associations with Mental Health in 3052 US Adults. Int. J. Environ. Res. Public. Health.

[17] Stanton R., To Q.G., Khalesi S., Williams S.L., Alley S.J., Thwaite T.L., Fenning A.S., Vandelanotte C. (2020). Depression, Anxiety and Stress during COVID-19: Associations with Changes in Physical Activity, Sleep, Tobacco and Alcohol Use in Australian Adults. Int. J. Environ. Res. Public. Health.

[18] Gallo L.A., Gallo T.F., Young S.L., Moritz K.M., Akison L.K. (2020). The Impact of Isolation Measures Due to COVID-19 on Energy Intake and Physical Activity Levels in Australian University Students. Nutrients.

[19] Factors Associated with Mental Health Disorders Among University Students in France Confined during the COVID-19 Pandemic|Adolescent Medicine|JAMA Network Open|JAMA Network. https://jamanetwork.com/journals/jamanetworkopen/fullarticle/2772154.

[20] Škrlec I., Talapko J., Pustijanac E., Meštrović T. (2021). Depression, Anxiety, Stress and Physical Activity in Health-Related University Students during COVID-19. Med. Sci. Forum.

[21] Srivastav A.K., Sharma N., Samuel A.J. (2021). Impact of Coronavirus Disease-19 (COVID-19) Lockdown on Physical Activity and Energy Expenditure among Physiotherapy Professionals and Students Using Web-Based Open E-Survey Sent through WhatsApp, Facebook and Instagram Messengers. Clin. Epidemiol. Glob. Health.

[22] Milić J., Škrlec I., Vranješ I.M., Podgornjak M., Heffer M. (2019). High Levels of Depression and Anxiety among Croatian Medical and Nursing Students and the Correlation between Subjective Happiness and Personality Traits. Int. Rev. Psychiatry.

[23] Nabkasorn C., Miyai N., Sootmongkol A., Junprasert S., Yamamoto H., Arita M., Miyashita K. (2006). Effects of Physical Exercise on Depression, Neuroendocrine Stress Hormones and Physiological Fitness in Adolescent Females with Depressive Symptoms. Eur. J. Public Health.

[24] Jin P. (1992). Efficacy of Tai Chi, Brisk Walking, Meditation, and Reading in Reducing Mental and Emotional Stress. J. Psychosom. Res..

[25] Henry J.D., Crawford J.R. (2005). The Short-Form Version of the Depression Anxiety Stress Scales (DASS-21): Construct Validity and Normative Data in a Large Non-Clinical Sample. Br. J. Clin. Psychol..

[26] Wang X., Hegde S., Son C., Keller B., Smith A., Sasangohar F. (2020). Investigating Mental Health of US College Students During the COVID-19 Pandemic: Cross-Sectional Survey Study. J. Med. Internet Res..

[27] Szabó M. (2010). The Short Version of the Depression Anxiety Stress Scales (DASS-21): Factor Structure in a Young Adolescent Sample. J. Adolesc..

[28] Benefits of Exercise for the Treatment of Depression—PubMed. https://pubmed.ncbi.nlm.nih.gov/2192427/.

[29] Scully D., Kremer J., Meade M.M., Graham R., Dudgeon K. (1998). Physical Exercise and Psychological Well Being: A Critical Review. Br. J. Sports Med..

[30] Long B.C., van Stavel R. (1995). Effects of Exercise Training on Anxiety: A Meta-Analysis. J. Appl. Sport Psychol..

[31] Lambert M.J., Bergin A.E., Garfield S.L. (1994). The Effectiveness of Psychotherapy. Encycl. Psychother..

[32] Huang J., Zheng Y., Gao D., Hu M., Yuan T. (2019). Effects of Exercise on Depression, Anxiety, Cognitive Control, Craving, Physical Fitness and Quality of Life in Methamphetamine-Dependent Patients. Front. Psychiatry.

[33] Oliveira-Silva I., Silva V.A., Cunha R.M., Foster C. (2018). Autonomic Changes Induced by Pre-Competitive Stress in Cyclists in Relation to Physical Fitness and Anxiety. PLoS ONE.

[34] Kandola A., Stubbs B. (2020). Exercise and Anxiety. Adv. Exp. Med. Biol..

[35] Fang Y.-Y., Huang C.-Y., Hsu M.-C. (2019). Effectiveness of a Physical Activity Program on Weight, Physical Fitness, Occupational Stress, Job Satisfaction and Quality of Life of Overweight Employees in High-Tech Industries: A Randomized Controlled Study. Int. J. Occup. Saf. Ergon. JOSE.

[36] Neumann R.J., Ahrens K.F., Kollmann B., Goldbach N., Chmitorz A., Weichert D., Fiebach C.J., Wessa M., Kalisch R., Lieb K. (2022). The Impact of Physical Fitness on Resilience to Modern Life Stress and the Mediating Role of General Self-Efficacy. Eur. Arch. Psychiatry Clin. Neurosci..

[37] Wood C.J., Clow A., Hucklebridge F., Law R., Smyth N. (2018). Physical Fitness and Prior Physical Activity Are Both Associated with Less Cortisol Secretion during Psychosocial Stress. Anxiety Stress Coping.

[38] Mücke M., Ludyga S., Colledge F., Gerber M. (2018). Influence of Regular Physical Activity and Fitness on Stress Reactivity as Measured with the Trier Social Stress Test Protocol: A Systematic Review. Sport. Med..

[39] Fillingim R.B., Blumenthal J.A. (1993). The Use of Aerobic Exercise as a Method of Stress Management. Princ. Pract. Stress Manag..

[40] Blumenthal J.A., Fredrikson M., Kuhn C.M., Ulmer R.L., Walsh-Riddle M., Appelbaum M. (1990). Aerobic Exercise Reduces Levels of Cardiovascular and Sympathoadrenal Responses to Mental Stress in Subjects without Prior Evidence of Myocardial Ischemia. Am. J. Cardiol..

[41] Blumenthal J.A., Madden D.J. (1988). Effects of Aerobic Exercise Training, Age, and Physical Fitness on Memory-Search Performance. Psychol. Aging.

[42] Sherwood A., Light K.C., Blumenthal J.A. (1989). Effects of Aerobic Exercise Training on Hemodynamic Responses during Psychosocial Stress in Normotensive and Borderline Hypertensive Type A Men: A Preliminary Report. Psychosom. Med..

[43] Hair J.F., Matthews L.M., Matthews R.L., Sarstedt M. (2017). PLS-SEM or CB-SEM: Updated guidelines on which method to use. Int. J. Multivar. Data Anal..

[44] Krejcie R.V., Morgan D.W. (1970). Determining sample size for research activities. Educ. Psychol. Meas..

[45] Lovibond P.F., Lovibond S.H. (1995). The Structure of Negative Emotional States: Comparison of the Depression Anxiety Stress Scales (DASS) with the Beck Depression and Anxiety Inventories. Behav. Res. Ther..

[46] Abadie B.R. (1988). Construction and Validation of a Perceived Physical Fitness Scale. Percept. Mot. Ski..

[47] Arrieta H., Rezola-Pardo C., Zarrazquin I., Echeverria I., Yanguas J.J., Iturburu M., Gil S.M., Rodriguez-Larrad A., Irazusta J. (2018). A Multicomponent Exercise Program Improves Physical Function in Long-Term Nursing Home Residents: A Randomized Controlled Trial. Exp. Gerontol..

[48] Nunnally J.C. (1994). Psychometric Theory 3E.

[49] Bray S.R., Born H.A. (2004). Physical Activity and Transition to University: Implications for Health and Psychological Well-Being. J. Am. Coll. Health.

[50] Brodani J., Kovacova N. (2019). The Interaction of Physical Activity, Joy of Movement and Quality of Life of High School Students at Different Ages. Phys. Act. Rev..

[51] Mandolesi L., Polverino A., Montuori S., Foti F., Ferraioli G., Sorrentino P., Sorrentino G. (2018). Effects of Physical Exercise on Cognitive Functioning and Well-being: Biological and Psychological Benefits. Front. Psychol..

[52] Miller B.M., Bartholomew J.B., Springer B.A. (2005). Post-Exercise Affect: The Effect of Mode Preference. J. Appl. Sport Psychol..

[53] Macías M.R., Robles M.T.A., Fuentes-Guerra F.J.G. (2021). Effects of Sport Teaching on Students’ Enjoyment and Fun: A Systematic Review and Meta-Analysis. Front. Psychol..

[54] Kwon S., Menezes A.M.B., Ekelund U., Wehrmeister F.C., Gonçalves H., da Silva B.G.C., Janz K.F. (2022). Longitudinal Change in Physical Activity and Adiposity in the Transition from Adolescence to Early Adulthood: The 1993 Pelotas Cohort Study. Int. J. Behav. Nutr. Phys. Act..

[55] Arnett J.J. (2000). A Theory of Development from the Late Teens through the Twenties. Am. Psychol..

[56] Gall T.L., Evans D.R., Bellerose S. (2000). Transition to First-Year University: Patterns of Change in Adjustment across Life Domains and Time. J. Soc. Clin. Psychol..

[57] Maher J.P., Doerksen S.E., Elavsky S., Hyde A.L., Pincus A.L., Ram N., Conroy D.E. (2013). A Daily Analysis of Physical Activity and Satisfaction with Life in Emerging Adults. Health Psychol..

[58] Hair J.F., Black W.C., Babin B.J., Anderson R.E. (2014). Multivariate Data Analysis: Pearson New International Edition.

[59] Raedeke T.D. (2007). The Relationship between Enjoyment and Affective Responses to Exercise. J. Appl. Sport Psychol..

[60] Byrne B.M. (2013). Structural Equation Modeling with LISREL, PRELIS, and SIMPLIS: Basic Concepts, Applications, and Programming.

[61] Kecojevic A., Basch C.H., Sullivan M., Davi N.K. (2020). The Impact of the COVID-19 Epidemic on Mental Health of Undergraduate Students in New Jersey, Cross-Sectional Study. PLoS ONE.

[62] Schlichtiger J., Brunner S., Steffen J., Huber B.C. (2020). Mental Health Impairment Triggered by the COVID-19 Pandemic in a Sample Population of German Students. J. Investig. Med. Off. Publ. Am. Fed. Clin. Res..

[63] Maher J.P., Hevel D.J., Reifsteck E.J., Drollette E.S. (2021). Physical Activity Is Positively Associated with College Students’ Positive Affect Regardless of Stressful Life Events during the COVID-19 pandemic. Psychol. Sport Exerc..

[64] Knipe D., Maughan C., Gilbert J., Dymock D., Moran P., Gunnell D. (2018). Mental Health in Medical, Dentistry and Veterinary Students: Cross-Sectional Online Survey. BJPsych Open.

[65] Donker T., van Straten A., Marks I., Cuijpers P. (2011). Quick and Easy Self-Rating of Generalized Anxiety Disorder: Validity of the Dutch Web-Based GAD-7, GAD-2 and GAD-SI. Psychiatry Res..

[66] Reitz A.K., Luhmann M., Bleidorn W., Denissen J.J. (2022). Unraveling the Complex Relationship between Work Transitions and Self-Esteem and Life Satisfaction. J. Pers. Soc. Psychol..

[67] Bryman A., Cramer D. (2012). Quantitative Data Analysis with IBM SPSS 17, 18 & 19: A Guide for Social Scientists.

[68] Tabachnick B.G., Fidell L.S., Ullman J.B. (2007). Using Multivariate Statistics.

[69] Saad S.K., Elshaer I.A. (2017). Organizational Politics and Validity of Layoff Decisions: Mediating Role of Distributive Justice of Performance Appraisal. J. Hosp. Mark. Manag..

[70] Aucejo E.M., French J., Araya M.P.U., Zafar B. (2020). The Impact of COVID-19 on Student Experiences and Expectations: Evidence from a Survey. J. Public Econ..

[71] Frontiers|COVID-19 and Distance Learning: Effects on Georgia State University School of Public Health Students. https://www.frontiersin.org/articles/10.3389/fpubh.2020.576227/full.

[72] Zayed M.A., Elshaer I.A. (2022). Physical Activities and Learning Experience of Higher Education Students: Mediating Role of Quality of Life and Physical Self-Esteem. Int. J. Environ. Res. Public. Health.

[73] Podsakoff N.P., LePine J.A., LePine M.A. (2007). Differential Challenge Stressor-Hindrance Stressor Relationships with Job Attitudes, Turnover Intentions, Turnover, and Withdrawal Behavior: A Meta-Analysis. J. Appl. Psychol..

[74] Elshaer I.A., Augustyn M.M. (2016). Testing the Dimensionality of the Quality Management Construct. Total Qual. Manag. Bus. Excell..

